# Synthesis and Characterization of a Composite Anion Exchange Membrane for Water Electrolyzers (AEMWE)

**DOI:** 10.3390/membranes13010109

**Published:** 2023-01-14

**Authors:** Somayyeh Rakhshani, Rodolfo Araneo, Andrea Pucci, Antonio Rinaldi, Chiara Giuliani, Alfonso Pozio

**Affiliations:** 1 Department of Astronautical, Electrical and Energy Engineering, University of Rome, Via Eudossiana 18, 00184 Rome, Italy; 2 Department of Chemistry and Industrial Chemistry, University of Pisa, Via Moruzzi 13, 56124 Pisa, Italy; 3 ENEA, C.R. Casaccia, Via Anguillarese 301, 00123 Rome, Italy

**Keywords:** anion exchange membrane, alkaline water electrolysis, membrane characterization

## Abstract

Anion exchange membranes (AEM) have gained attention recently as a promising candidate for low-cost water electrolysis systems to produce hydrogen, linked with renewable energy resources as a sustainable alternative to fossil fuels. The development of potential materials for producing and analyzing AEM is an imperative step towards commercialization and plays a competitive role in the hydrogen production industry. In this article, we developed a composite anion exchange membrane prepared by activating a commercial support structure (Celgard^®^ 3401) with a commercially available functional group (Fumion^®^ FAA-3) through a phase-inversion process. Fourier-transform infrared spectroscopy (FTIR) and Scanning Electron Microscopy (SEM) analysis demonstrated the phase-inversion procedure as an effective methodology. Furthermore, the cell performance test result (with Celgard/Fumion) was very promising and even better in comparison with a commercial membrane commonly applied in alkaline electrolysis (Fumasep). We also developed a testing procedure for membrane performance evaluation during electrolysis which is very critical considering the effect of CO_2_ absorption on membrane conductivity.

## 1. Introduction

As we face a major challenge in the energy and environment sectors, considering the anthropogenic climate change hypothesis, searching for alternative energy strategies has become critical for future world energy demands. Among the numerous alternative energy approaches, green hydrogen serves as a low-cost, easily available, and abundant energy source with zero emissions of carbonaceous species and can play a key role in the energy transition strategy toward a stable and green future as it has a significant role in several applications and industries [1,2]. At the same time, it should be taken into consideration that the footprint of hydrogen technologies depends on their production processes and energy sources [3]. From this point of view, water electrolysis is a very promising and desired approach because of its compatibility with different types of electricity generation [4,5]. The search for investigation of efficient and cost-effective anion exchange membrane electrolyzers have been given much attention due to their ability to offer the advantages of conventional alkaline electrolysis technology [6,7] while also addressing the issue of cost associated with proton exchange membranes (PEMs) by using non-platinum group metals as electrocatalysts [8].

The anion exchange membrane water electrolyzer (AEMWE) is a technology built in a stack with solid polymer membranes to provide high volumetric energy density and it is compatible with non-precious metal catalyst electrodes which results in cost reduction of hydrogen production. The main difference between alkaline water electrolysis (AWE), which is a well-developed and mature technology [9,10,11], and AEMWE is that the diaphragm that separates two half-cells in the former technology, despite being permeable to ions between two sides of the system, is not conductive. The conductivity is provided by alkaline hydroxide filling a porous diaphragm, while in AEMWE, the polymeric membrane is non-porous and has intrinsic anionic conductivity [1,12,13,14,15]. It is worth mentioning that in contrast to PEM electrolyzers which use only polymer electrolytes, many AEMWEs use liquid electrolytes (e.g., KOH or K_2_CO_3_ solutions) in addition to polymer electrolytes to improve reaction kinetics [16]. The liquid electrolyte increases the local pH at the catalyst–electrolyte interface, generating an additional electrochemical interface. Having addressed the major challenges associated with AWEs, AEMWEs have attracted increasing interest from industrial and academic researchers in recent years.

Numerous studies and several papers and reviews have been published on the development of alkaline membranes for different applications (electrodialysis, electrodialysis reversal, desalination, or electro-deionization [17,18]) and from different aspects (separate components, material, technology, and operational conditions [2,16,19,20,21]). Most of the membranes produced were optimized for chemically less-aggressive environments concerning the pH and the operating temperature. The literature concerning the synthesis of anion exchange membranes (AEM) regarding those that use a hydroxide exchange membrane specifically designed for the electrolysis of water is scarce. The main function of the anionic membrane in alkaline electrolyzers is to transport hydroxide ions from the cathode to the anode and to avoid the movement of electrons and crossover of the gases produced by the electrochemical reactions in the two compartments. These membranes should ideally have high OH^−^ conductivity, high chemical stability (alkaline, oxidative, and thermal stability), excellent mechanical properties under wet conditions and high differential pressures, and low gas permeability. Generally, such membranes consist of a polymeric backbone that is responsible for mechanical and thermal stability and that is modified by positively charged functional groups on their backbone or pendant side chains. These modifiers facilitate anion movement and are responsible for ion-exchange capacity, ion conductivity, and transport number, enabling a zero-gap configuration and differential pressure operation.

A variety of AEM backbones have been studied and developed so far, such as polystyrene [22,23], poly-phenylene oxide (PPO) [24,25], polysulfone [26] or fluorinated polymers [27,28,29], poly-ethylene-co-tetrafluoroethylene (ETFE) [30], polyetherimide (PEI) [31], polyvinyl alcohol (PVA) [32], poly-ether-ether ketone [33], poly(arylene ether ketone) [34], and poly-carbazole [35]. As well, the most extensively investigated functional groups are: –NH_3_^+^, –RNH_2_^+^, –R_2_NH^+^, =R_2_N^2+^, –R_3_P^+^, –R_2_S^+^, quaternary ammonium salts (QA) [36,37], and tertiary amines (e.g., BTMA) [31,32]. The cycloaliphatic QAs show remarkable alkaline stability [38].

As investigated and reported [6,39,40], the major challenge of anion exchange membranes is their low chemical and thermal stability, since the backbone and the functional group both suffer from hydroxide-induced degradation under alkaline conditions [41]. The degradation of polymer backbones leads detrimentally to chain rift, decreases the molecular weight, and results in increased brittleness of the membrane, and this effect is prominent in the presence of aromatic ether groups. Therefore, aromatic ether groups, which are present in many cheap and easily accessible polymers such as PEEK, PESU, and polyphenylene oxide (PPO), should be avoided. Furthermore, anion exchange membranes suffer from lower ionic conductivity in comparison to PEMs (such as Nafion) due to the lower mobility of OH^−^ than that of H^+^.

Currently, a few alkaline solid polymeric membranes have been commercialized, such as Fumasep^®^, Sustainion^®^, Aemion^TM^, and Orion^TM^, and their main characteristics are summarized in Table 1 [2,39]. Membranes with different backbone materials show different conductivity, as shown in Table 1. The last three have a very high value with respect to Fumasep^®^, but it should be kept in mind that Fumasep is activated in Cl^−^ form, which has inferior mobility to that of OH^−^ [42], with a significantly higher thickness. There are very sporadic literature data on the usage of these membranes, and many were obtained with unequal activation procedures or ionomer material, cell components, operational conditions, and even time periods of the cell performance tests, which makes a comparative study very difficult. The most-used membrane that has shown stable performance in AEMWE [1,43] is Fumasep, which we used as reference material in this article.

The activation of porous support with desirable mechanical characteristics is a very simple approach to synthesizing anionic membranes. The performance of the membrane is then profoundly influenced by the supporting structure and the ionomer coupled together, so investigating the material and techniques to fabricate the backbone and activate it with different ionomers is a very interesting approach to developing AEMs. A supporting structure that can be used for this purpose is Celgard^®^, a typical polypropylene (PP) microporous membrane separator that is recognized for use in batteries [47,48]. PP is a low-cost polymer that is widely used for microporous membrane fabrication globally because of significant properties such as good thermal and mechanical stability and high resistance to extreme pH conditions [43,44]. Due to its numerous manufacturing applications and the huge amount of PP used globally, much research has been conducted on the sustainable assessment of PP production and recycling recently [49]. In the past, Stano et al. [50] used Celgard 2400 membranes modified with graft polymerization of acrylic acid after plasma activation in the conventional liquid alkaline electrolyzer. Tsehaye et al. [51] used Celgard 3501 impregnated with a commercial anion exchange ionomer (Fumion) to modify the support membrane to use as a separator in a Zn–air battery.

In this work, a composite anion exchange membrane was prepared by activating a porous support of commercial Celgard 3401 with an ionomer solution in a phase-inversion process. Phase inversion is an established technique commonly used for preparing membrane nano/microstructures. It is referred to as a chemical process in which a polymer is transformed from a liquid state to a solid state [48,52]. We used the commercially available ionomer solution Fumion (Fumatech Co., Bietigheim-Bissingen, Germany) with a QA functional group. The chemical structure of the Fumion polymer has not been disclosed; however it is reported [53] to contain a polyaromatic polymer with quaternary ammonium functional groups. The composite membrane was synthesized, characterized, and successively tested in a small lab electrolyzer to evaluate its performance. With the promising results revealed, we approach the challenge of fabricating porous substrates with controlled properties and a desired structure using low-cost polymeric materials with a highly efficient method to provide a robust, reproducible backbone for AEM in the future.

## 2. Experimental Procedures

### 2.1. Preparation of the Composite Anion Exchange Membranes

**Figure 1:**
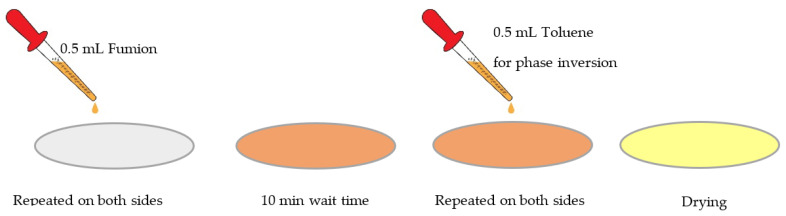
Schematic of membrane activation procedure in the phase-inversion method.

The composite anionic membrane reported here was prepared using a microporous monolayer membrane made of polypropylene with 41% porosity and 25 µm thickness, with surfactant-coated Celgard 3401 (Celgard^®^, Charlotte, CA, USA) to improve its hydrophilicity as the backbone, and was activated with Fumion FAA-3 solution in NMP 10% (Fumatech, Bietigheim-Bissingen, Germany). Activation was undertaken to ensure ion-exchange capacity and hydroxide conductivity for AEM applications. We carried out the activation on small pieces of porous Celgard (4 cm × 4 cm). Our application relies on phase inversion, which involves the polymer transforming from the liquid phase of the solution into the solid phase. The concept of the phase-inversion process has been explained elsewhere [52]. Phase inversion can be induced with different techniques, such as immersion precipitation, precipitation by controlled evaporation, thermal precipitation, or vapor-phase precipitation. The choice of method of phase inversion is highly dependent on the type of polymer and the solvent used to dissolve the polymer. As the first approach, we decided to use immersion precipitation by customizing it into a manual batch mode, applying a certain amount of ionomer solution and inversion solution. For each piece of the sample, we dropped 0.5 mL of Fumion FAA-3 solution on the membrane surface until it became transparent, and then we did the same on the other side of the membrane. After 10 min, we dropped 0.5 mL of toluene—as a non-solvent to induce inversion—on the Celgard surface. After 10 min, we repeated the operation on the other side of the membrane. Then we let it dry at room temperature overnight under the hood (Figure 1).

### 2.2. Characterization

#### 2.2.1. Physical Chemical Analysis

Fourier Transform Infrared (FTIR) spectra of dried samples were collected in the wave number range 400–3500 cm^−1^ using a Nicolet iS5 FT-IR spectrometer (Thermo Fisher Scientific, Waltham, MA, USA) equipped with an attenuated total reflectance (ATR) accessory. The measurements were recorded using a diamond crystal cell ATR, typically using 32 scans at a resolution of 4 cm^−1^. The samples were all measured under the same mechanical force pushing the samples into contact with the diamond crystal. No ATR correction has been applied to the data. SEM morphological characterization was acquired with a field-emission gun scanning electron microscope (ZEISS, Jena, Germany). The air permeability measurement of the membrane after activation with Fumion was performed with a Gurley 4320 densitometer (Rycobel, Deerlijk, Belgium) as described elsewhere [54].

#### 2.2.2. Ionic Exchange Capacity (IEC)

The IEC of an ion-exchange membrane (IEM) is an analytical parameter that represents the ability of ionic transfer within an alkaline solid polymeric membrane and is significantly influenced by the ion conductivity of the membrane [3]. It is measured when the counter-ions are OH^−^ using a titration method and it is expressed as milliequivalent per gram of the dry membrane (meq g^−1^ dry IEM). The method is well-described elsewhere [55]. Briefly, the membrane was converted into OH^−^ form by immersing it in 100 mL 1M KOH and 2 days. Then the membrane was washed and immersed in 100 mL of a 0.01 HCl solution for 24 h and finally we measured IEC using back titration. The OH^−^ content in the membrane was calculated using the following equation:

(1) $$IEC=\frac{n_{HCL,i}-n_{HCL,f}}{m}$$

where $n_{HCL,i}$ is the amount of HCl in the initial solution (0.01 M, 100 mL), $n_{HCL,f}$ is the amount of HCl in the final solution after neutralization of the membrane, and *m* is the dry membrane mass. Attention must be placed in this characterization that could be affected in some step by the accumulation of carbonate and bicarbonate ions with an underestimation of the final value of IEC.

#### 2.2.3. Ionic Conductivity

**Figure 2:**
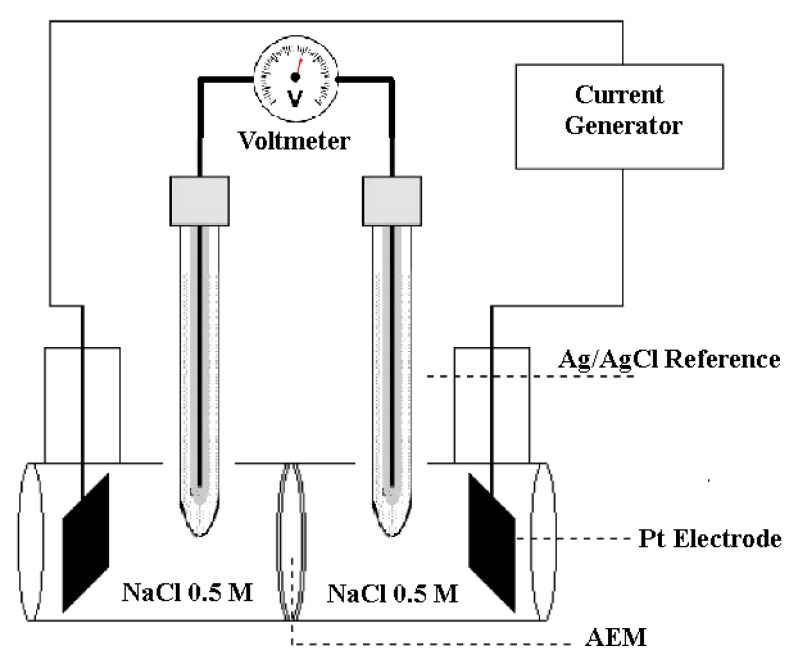
Schematic representation of experimental conductivity cell.

The membrane conductivity can be obtained from the measurement of the resistivity of the membrane against the flow of current using a four-point probe electrochemical cell (Figure 2). The method is explained elsewhere [55]. Briefly, the cell is composed of two compartments that can house membranes with a diameter of 3.5 cm with an area exposed to the solution equal to 7.07 cm^2^. The ionic conductivity of the membrane was determined with at least three successive measurements of the potential difference between the reference electrodes. A linear voltage sweep was applied in short time intervals (5 s), voltage vs. current was plotted, and the slope gives us the resistance (Ω) without the membrane and with the membrane between the two compartments. For this experiment, the NaCl solution in the cell was maintained at 0.5 M. Without the membrane, the measured resistance of the aqueous NaCl solution between the two reference electrodes is *R_cell_*; with a membrane, the total resistance is the sum of the resistance of the cell and that of the membrane: $R_{measure}=R_{membrane}+R_{Cell}.$ Knowing the cell resistance (*R_cell_*), the value of the membrane resistance is defined as the difference between these two resistances: $R_{membrane}=R_{measure}-R_{Cell}$. The ionic conductivity, *σ* (S cm^−1^), of the membrane can be determined using the following relationship:

(2) $$\sigma =\frac{1}{R_{membrane}}\frac{l}{S}$$

where *l* is the thickness of the membrane (cm), *S* is the surface area of the membrane exposed to the electric field (cm^2^), and *R_membrane_* is the membrane resistance (Ω). The product *R_membrana_* × *S* represents the area-specific resistance (ASR).

#### 2.2.4. Electrochemical Performance

**Figure 3:**
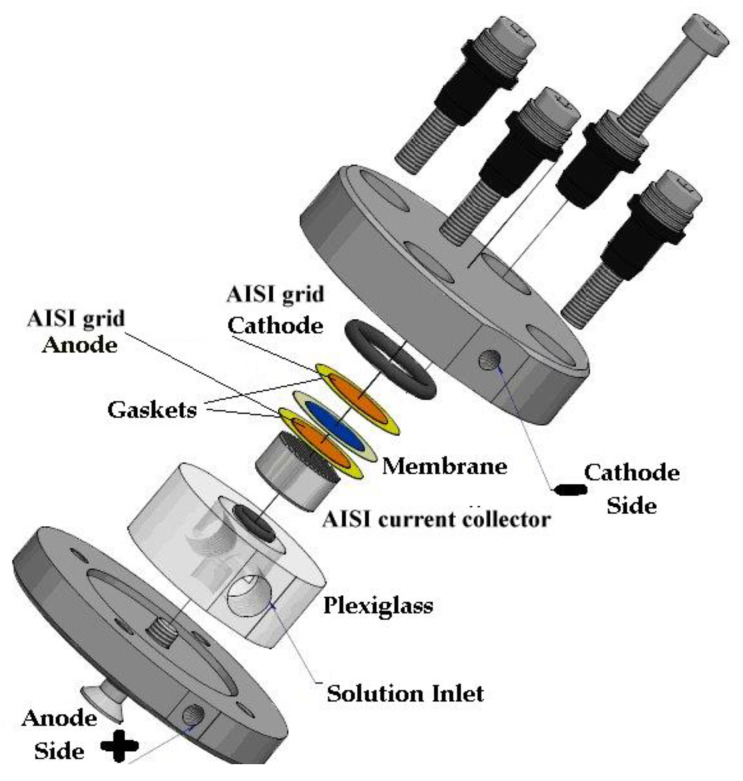
Experimental cell for electrochemical measurement.

The electrochemical performance test of the membrane was carried out in a lab-scale 2 cm^2^ electrolyzer (two-electrode) at room temperature with stainless steel gas diffusion electrodes (GDE). The cell (Figure 3) was made of steel on both the cathode and anode sides. The GDE (Ø 16 mm) that ensured the electrical contact of the anionic membrane and the diffusion of the water and gases produced was supplied by Bekaert Fiber Technologies (Belgium). It is a commercial matrix in sintered metal fiber in AISI 316-L steel with a thickness of 0.51 mm and with a porosity of 82% and compressibility of approx. 5.4%. The anodic side can be fed with distilled water or a KOH solution 0.5 M (in our case study the latter was the choice), contained in a 500 mL polyethylene tank using a metering pump (KMS), in PTFE with a flow of 100 mL min^−1^. The cathode side output can be connected to a volumetric system for measuring the hydrogen produced.

The cell was connected to a Solartron Mod. 1287 potentiostat/galvanostat and a Solartron Mod. 1260 frequency response analyzer, both interfaced with a GPIB card to a personal computer. Electrochemical impedance spectroscopy (EIS) measurements were performed in the 1 MHz-1 Hz frequency range at open circuit potential (OCP); the amplitude of the AC signal was always 10 mV_pp_. Both static and dynamic galvanic polarization were performed in the same cell configuration as the EIS measurements. The characteristic E vs. I curves were also recorded on the cell at room temperature (298 °K) in the current range of 0–1000 mA cm^−2^. Graphs of cell voltage versus time were recorded continuously and impedance spectra were periodically acquired at the OCP.

EIS is a quantity that measures the opposition of a system to the passage of current and in DC conditions, coincides with the resistance value. It is also a “transient” technique, in the sense that, when one of the quantities that determine the state of the system is perturbed, relaxation to a new steady state occurs with a speed dependent on the parameters (kinetics, transport, etc.) of the system.

The impedance spectra can be represented with Nyquist diagrams, in which the high-frequency intercept (R_HF_) represents the ohmic resistance of the system, while the diameter of the semicircle is basically related to the polarization resistance sum of various contributions (diffusion, charge transfer).

The measurements were conducted on the electrolysis cell shown in Figure 3 in a flow of 0.5 M KOH at a temperature of 25 °C. The measured resistance value R_HF_ is that relating to the high-frequency resistance, i.e., the intercept with the Z_Re_ axis by Z_Imm_ = 0 in the Nyquist diagram. Once the value is known, it is possible to calculate the specific resistance R_Ω_ (Ω cm^2^) for the cell:

(3) $$R_{\Omega }=R_{HF}\times S$$

where S is the surface area of the membrane (2.0 cm^2^) in the electrolyzer. This measurement also allows estimation of the intrinsic conductivity of the membrane (i.e., the conductivity induced by counter ions associated with positive ionic sites of the ionomer and favoured by water molecules).

## 3. Results and Discussion

### 3.1. FTIR and SEM Membrane Analysis

**Figure 4:**
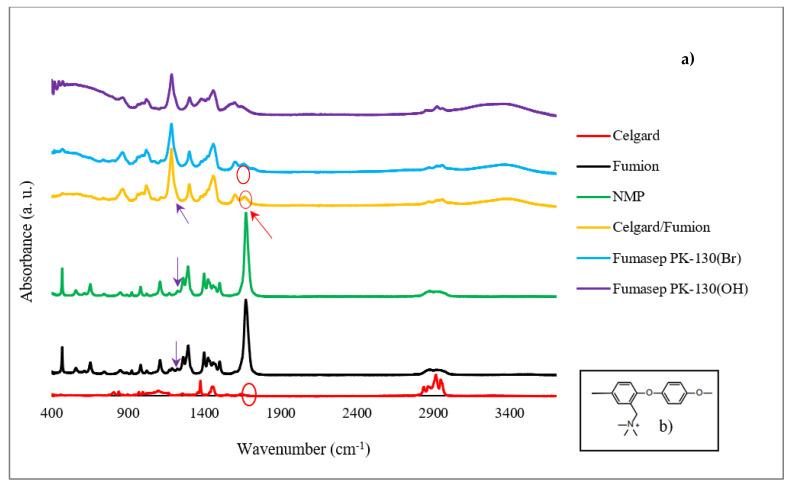
(**a**) FTIR spectrum of the Celgard before and after activation, Fumion, Fumasep PK-130 (in Br^−^ and OH^−^ form), and NMP solvent, (**b**) chemical structure of Fumion.

The FTIR analysis confirms the success of the Celgard activation with the ionomer solution by applying the proposed procedure. FTIR spectra of the Celgard samples before and after the Fumion activation are reported in Figure 4. Fumion (ionomer solution in NMP solvent) and NMP are also reported for clarity. Fumasep PK-130, in the Br^−^ and OH^−^ form, is used as a reference. Chemically, the Fumion ionomer is a polyaromatic polymer with ether bonds in the main chain and quaternary ammonium groups attached to the main chain (Figure 4b). The spectrum of the membrane after the ionomer activation (Celgard/Fumion) shows the presence of some peaks due to the ionomer. In particular, it is possible to observe the infrared absorption at 1671 cm^−1^, due to the =CH_2_ bending vibration from the aromatic rings, and at 1602 cm^−1^, attributable to the stretching vibration of the N-C groups from the quaternary ammonium groups. In addition, the peak at 1187 cm^−1^ is detected in both the Celgard/Fumion (intense peak) and Fumion spectra (weak peak). This infrared absorption is not present in the NMP spectrum. This evidence suggests that the peak is probably due to the polymer dissolved in the ionomer solution. It is worth noting that the FTIR spectrum of Celgard activated with Fumion is comparable to that of the commercial membrane Fumasep, selected as the reference.

**Figure 5:**
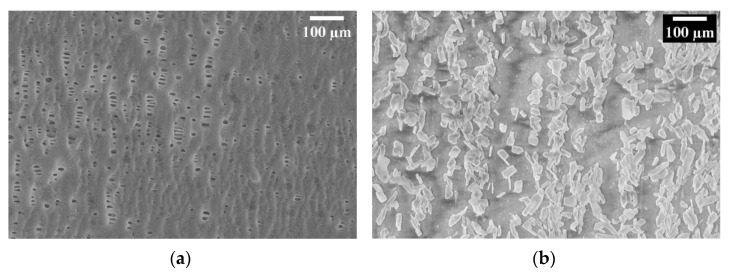
SEM image of Celgard 3401, (**a**) before activation (**b**) after activation with Fumion.

The Celgard support consists of intertwined fibers with a porosity of about 41% and a variable pore size on the order of microns or smaller (Figure 5a). As visible in Figure 5b, after activation, the Fumion ionomer filled the pores, and we can see they are aligned along the pores’ locations which confirms the presence of the functional QA group in our composite membrane.

The permeability measurements on the activated Celgard support, which had a Gurly number of 620 s initially, were infinite time after activation with Fumion. This means the pores had been filled with Fumion. Furthermore, it was subsequently put in the conductivity measurement cell in which one side of the activated membrane was filled with the solution and left overnight. No solution passed through the membrane to the other side, indicating that there were no pores left inside the membrane. Further analysis is necessary to better evaluate how the pore volume and porosity of the substrate affect the membrane’s properties, and their correlation with phase inversion results.

### 3.2. Ion-Exchange Capacity and Ionic Conductivity Measurement

**Figure 6:**
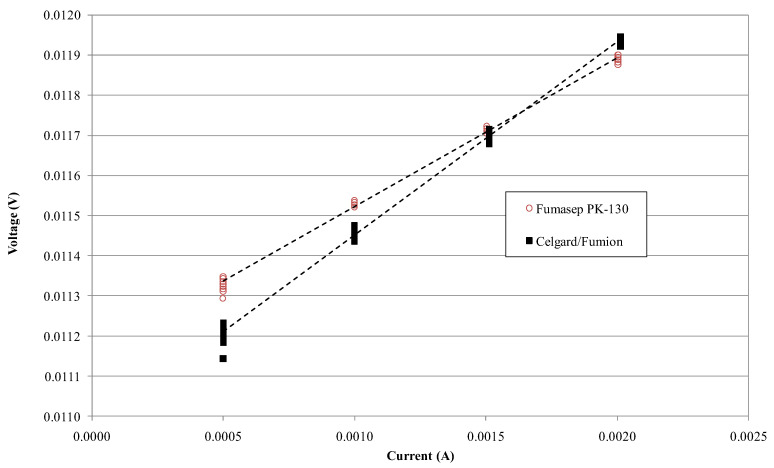
Voltage vs. current measurement on Fumasep-PK-130 (○) and Celgard/Fumion (■) membrane in NaCl 0.5 M at 25 °C.

Figure 6 shows the voltage–current measurement on Fumasep-PK-130 and Celgard/Fumion membranes. The slope is greater for Fumasep, indicating a higher resistance due to its thickness. Table 2 shows the calculated conductivity for both membranes using Equation (2), obtained by measuring K_cell_ and R_cell_ in a NaCl 0.5 M solution at 25 °C (respectively, K_cell_ = 369.39 cm^−1^, R_cell_ = 0.038 Ω). The conductivity of the commercial Fumasep is within the ranges provided by the producer in the datasheet for the Cl^−^ form (4–8 mS cm^−1^). Table 2 also reports IEC defined using titration for the samples. Despite the composite membrane having lower conductivity and IEC than the commercial one, the ASR is lower due to its minor thickness. This result represents a good compromise where poor ion conductivity could be compensated for with a low area-specific resistance (ASR).

### 3.3. Electrochemical Characterization of Membranes

To evaluate membrane functionality, long-term electrolysis tests were carried out on both membranes to allow comparison.

**Figure 7:**
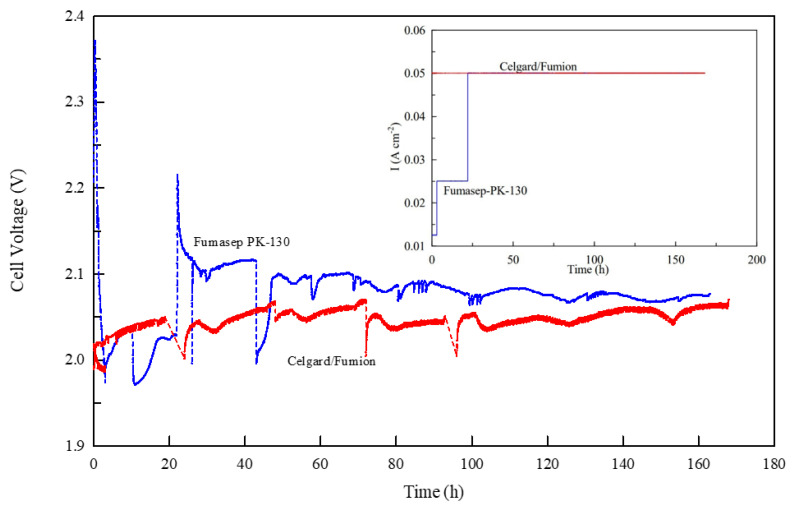
Measurement of cell voltage vs. time for Celgard/Fumion and Fumasep-PK-130 membranes at 25 °C. The inset figure shows the current density profile during the experiment.

Figure 7 shows the electrolysis voltage of the two cells at a current density of 50 mA cm^−2^ during the first 163 h of operation. Both membranes exhibit a voltage between 2 and 2.1 V and over time reach a value of about 2.05 V. However, a substantial difference is observed during the initial phase (the first 20 h) while the membranes were pre-treated the same way (approximately 24 h in KOH 0.5 M). As illustrated in the inside figure of Figure 7 the commercial Fumasep membrane cannot immediately maintain the electrolysis current set at 50 mA cm^−2^ and so it is necessary to reduce the initial current value to 12.5 mA cm^−2^ and then progressively increase it to 25 and finally to 50 mA cm^−2^. The behavior appears to be closely related to the conductivity of the membrane, which at first does not provide adequate transport of OH^−^ ions inside, more likely due to the formation of larger, less mobile ions (CO_3_^2−^ and HCO_3_^−^) due to the carbonation of the membrane.

This is explained by the fact that AEM is very reactive to CO_2_ in the environment. In an AEMWE, hydroxide anions (OH^−^) are responsible for creating current as they are transported through the anion exchange membrane (AEM) from the cathode to the anode side of the cell. However, upon exposure to the ambient air which contained CO_2_, the carbonation process takes place very fast following the chemical reactions (4) and (5) so that the accumulation of bicarbonate ions on the membrane interface confines the mass transport of OH^−^ ions at the cathode. Thus, hydroxide concentration in the membrane decreases and negatively affects cell performance [56,57,58].

(4) $$OH^{-}+CO_{2}↔HCO_{3}^{-}$$

(5) $$OH^{-}+HCO_{3}^{-}↔CO_{3}^{2-}+H_{2}O$$

This effect appears to be detrimental for several reasons: (1) The membrane is normally produced in chloride or bromide form and changed to OH^−^ form before insertion into the cell by immersion in an alkaline solution. (2) The carbonation process depends on the air-exposure time of the membrane after treatment with hydroxide and heavily influences its conductivity and electrolysis performance. (3) Over time the level of CO_2_ contamination of the circulating solution can vary, especially if the cell remains inactive for a long time. In the literature, methods have been reported for the de-carbonation of membranes before ex situ conductivity measurement. As an example, Kimura et al. [56] tested an activation procedure in a closed system with a CO_2_-free atmosphere using nitrogen-treated water. Ziv et al. [57] electrochemically purged the carbonate ions in the membrane by applying an electrolysis current in a humidified and CO_2_-free atmosphere for one week. In this way, the oxygen generated at the anode (6) replaces the CO_2_ and the hydroxide generated at the cathode replaces the carbonate ions by shifting reaction (4) to the left over time.

(6) $$2OH^{-}\to 1/2O_{2}+H_{2}O+2e^{-}$$

(7) $$2H_{2}O+2e^{-}\to H_{2}+2OH^{-}$$

**Figure 8:**
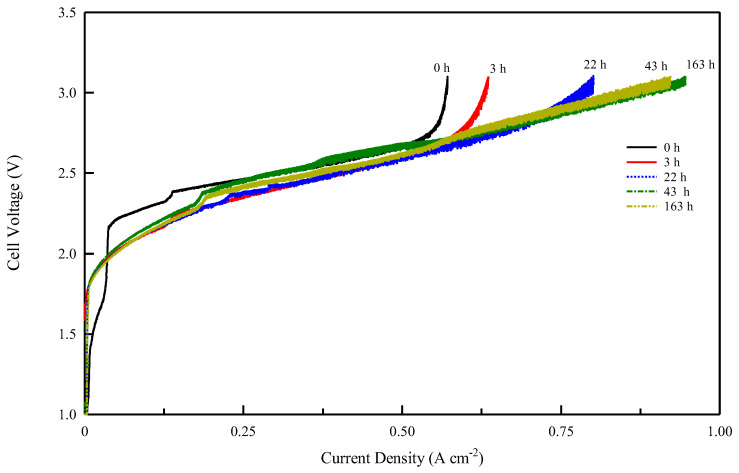
Cell voltage vs. current density profile for Celgard/Fumion membrane after different electrolysis times at 25 °C.

**Figure 9:**
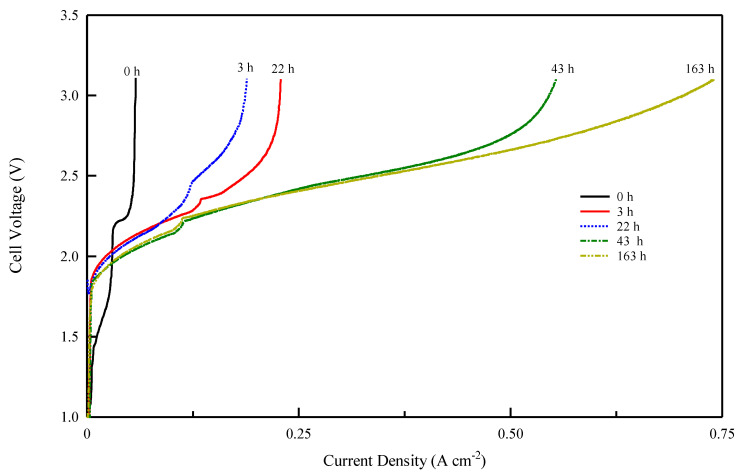
Cell voltage vs. current density profile for Fumasep-PK-130 membrane after different electrolysis times at 25 °C.

These studies show that to verify the performance of an anionic membrane, it is necessary to achieve a prolonged pre-treatment phase of electrolysis with a gradual increase in current to eliminate CO_2_. In our study, both membranes showed the same carbonation effect in electrolysis (Figure 7). Figure 8 and Figure 9 show the voltage vs. current density after the different electrolysis times shown in Figure 7. The electrolysis at constant current density was stopped after various time intervals and a Galvano-dynamic experiment was performed at 1 mAs^−1^. Initially, the electrolyzer’s current density is constrained by the conductivity drop due to the accumulation of carbonate and bicarbonate ions, which inhibit the transport of OH^−^ ions. Following Figure 8 and Figure 9, both systems reached a higher maximum current density as electrolysis proceeded but with different values. A minimum amount of current and gas evolution (reactions 6 and 7) allows the elimination of carbonate ions and the recovery of the OH^−^ conductivity resulting in an increase in the electrolysis current. However, the maximum current density achievable by the system progressively increases with the working hours of the electrolysis test. The same behavior of current limitation due to the CO_2_ effect was studied in detail by Ziv et al. in anion exchange membrane fuel cells (AEMFCs) and is well illustrated in references [57,58]

As follows from the voltage–current density plot, at zero time the cell with the Celgard/Fumion membrane reaches a maximum current density of 569 mA cm^−2^ while after 163 h of electrolysis the maximum current reaches 933 mAcm^−2^. The cell with Fumasep membrane also shows similar behavior, with 57 mAcm^−2^ at zero time and 740 mAcm^−2^ after 163 h. For both cells, the presence of a limiting effect due to hydroxide transport appears in the first working hours. However, the time expansion is more noticeable for the commercial membrane, whose recovery time is longer, likely due to its higher thickness. The results also indicate that thinner membranes are preferred since they react more rapidly to the de-carbonation process during electrolysis.

The results revealed that measuring membrane performance in a small electrolysis cell is very challenging, and to make a reliable comparison between different membranes requires long-term electrolysis. Furthermore, preparation using phase inversion is a fast and easily applicable method for synthesizing composite membranes with different backbone structures and ionomer solutions.

**Figure 10:**
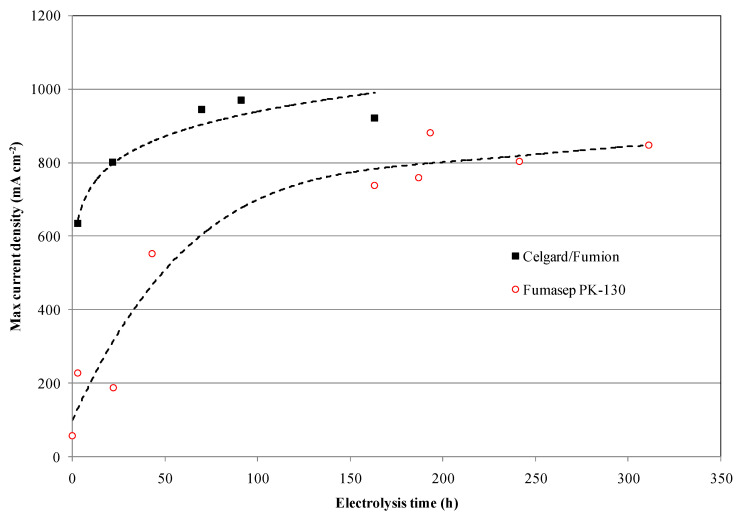
Maximum current density profile at 3.1 V vs. time on Fumasep-PK-130 and Celgard/Fumion at 25 °C.

**Figure 11:**
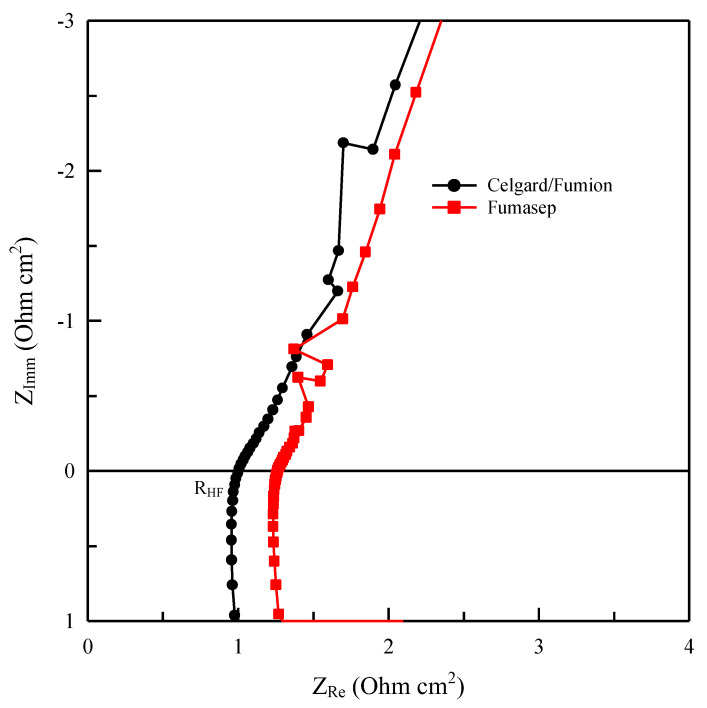
Nyquist plot for cell with Fumasep and Celgard/Fumion membranes at 25 °C and OCP (in electrochemical impedance spectroscopy—EIS).

Continuing the measurements, the commercial Fumasep membrane did not reach the same performance as the Celgard/Fumion, even after the electrolysis test was prolonged up to 310 h (Figure 10 and Figure 11).

Further insight into the membranes is provided by the impedance analysis of the two cells in hydroxide form during the electrolysis test. The conductivity value calculated from cell impedance spectroscopy has an average value of 5 ± 1 mScm^−1^ for Celgard/Fumion while it is 9 ± 1 mScm^−1^ for Fumasep. The in situ measurements confirm that despite the lower conductivity of our composite membrane, from an electrochemical standpoint it is more efficient thanks to the lower specific resistance due to its lower thickness. It also seems that the thickness of the membrane influences the time needed for activation of the membrane. The thinner the membrane is, the shorter the time needed for releasing carbonate ions, as observed for the composite membrane (Celgard/Fumion).

## 4. Conclusions

A composite anion exchange membrane was synthesized by applying commercial material—support structure Celgard 3401 and commercially available functional group Fumion FAA-3—through a phase-inversion process and successfully tested in an electrochemical cell. The success of phase inversion as a method of activation was demonstrated with FTIR and SEM analysis. This study showed that the composite membrane has a higher maximum current density than the Fumasep membrane. This implies better performance and supports the validity of this practice as a potential approach for fast production and characterization of AEMs which needs further optimization. In this regard, the idea of producing porous substrates with controlled rheological properties and desired structure out of low-cost polymeric material with an efficient technique to provide a robust, reproducible backbone for AEM stands out.

Moreover, we evaluated and developed a testing protocol for membrane performance during electrolysis, which is very relevant, considering the effect of CO_2_ absorption on conductivity, to create a reliable inter-laboratory comparison. The absence of a well-established international cell performance test protocol, however, seems to be a profound hurdle to enable researchers to make a solid comparison with literature results.

## Figures and Tables

**Table 1:** Commercial AEM characteristics.

Brand Name	Company Country	Product Code	Material	Thickness	IEC (meq/g)	Conductivity (mS cm^−1^)	Refs.
**Fumasep^®^**	Fumatech	FAA-3-PK-130	PK reinforced	130	1.1–1.4	4.0–8.0 (Cl^−^)	Data sheet
	(Germany)						
**Sustainion^®^**	Dioxide Material	37–50	Styrene based	50	-	70, 80 (OH^−^)	[14,44]
	(USA)						
**Aemion^TM^**	Ionomer Innovation Co. (Canada)	AF1-HNN8-	HMT-PMBI * [45]	50	2.1–2.5	80 (OH^−^)	Data sheet
		AF1-HNN5-		50	1.4–1.7	15–25 (OH^−^)	Data sheet
**Orion^TM^**	Orion Polymer	Orion TM1™	Polyphenylene [46]	30	2.1	60 (OH^−^)	Data sheet
	(USA)						

* HMT-PMBI: hexamethyl-*p*-terphenyl poly (bibenzimidazolium).

**Table 2:** IEC and conductivity of sample membranes.

Membrane	Thickness (μ)	IEC (meq g^−1^)	R_measure_ (Ω)	R_membrane_ (Ω)	ASR (Ω cm^2^)	σ_membrane_ (mS cm^−1^)
Celgard/Fumion	25	0.44	0.22	0.19	1.3	3.0 ± 0.1
Fumasep PK-130	130	1.29	0.36	0.32	2.3	5.7 ± 0.2

## Data Availability

The data presented in this study are available on request from the corresponding author.
